# Coffee intake, genetic variants, and chronic kidney disease: a cross-sectional analysis of the Japan Multi-Institutional Collaborative Cohort (J-MICC) study

**DOI:** 10.1007/s00394-025-03819-2

**Published:** 2025-10-15

**Authors:** Taichi Unohara, Ryosuke Fujii, Takeshi Watanabe, Akari Matsuura, Yuka Torii, Kahori Kita, Masashi Ishizu, Megumi Hara, Yuichiro Nishida, Mako Nagayoshi, Takashi Matsunaga, Rieko Okada, Yoko Kubo, Shiroh Tanoue, Yoshifumi Hidaka, Takeshi Nishiyama, Hiroko Nakagawa-Senda, Teruhide Koyama, Isao Watanabe, Kiyonori Kuriki, Naoyuki Takashima, Keiko Kondo, Masahiro Nakatochi, Yukihide Momozawa, Takashi Tamura, Keitaro Matsuo

**Affiliations:** 1 Department of Preventive Medicine, Tokushima University Graduate School of Biomedical Sciences, 3-18-15, Kuramoto-Cho, Tokushima, 770-8503 Japan; 2 Student Lab, Faculty of Medicine, Tokushima University, Tokushima, Japan; 3 Department of Preventive Medical Science, Fujita Health University School of Medical Sciences, 1-98 Dengakugakubo, Toyoake, Aichi 470-1192 Japan; 4 Department of Preventive Medicine, Nagoya University Graduate School of Medicine, Nagoya, Japan; 5 Department of Preventive Medicine, Faculty of Medicine, Saga University, Saga, Japan; 6 Department of Epidemiology and Preventive Medicine, Kagoshima University Graduate School of Medical and Dental Sciences, Kagoshima, Japan; 7 Department of Public Health, Nagoya City University Graduate School of Medical Sciences, Nagoya, Japan; 8 Department of Epidemiology for Community Health and Medicine, Kyoto Prefectural University of Medicine, Kyoto, Japan; 9 Laboratory of Public Health, Division of Nutritional Sciences, School of Food and Nutritional Sciences, University of Shizuoka, Shizuoka, Japan; 10 NCD Epidemiology Research Center, Shiga University of Medical Science, Otsu, Japan; 11 Public Health Informatics Unit, Department of Integrated Health Sciences, Nagoya University Graduate School of Medicine, Nagoya, Japan; 12 Laboratory for Genotyping Development, Center for Integrative Medical Sciences, RIKEN, Yokohama, Japan; 13 Division of Cancer Epidemiology and Prevention, Aichi Cancer Center Research Institute, Nagoya, Japan; 14 Department of Cancer Epidemiology, Nagoya University Graduate School of Medicine, Nagoya, Japan

**Keywords:** Chronic kidney disease, Coffee, Genetic variant, Caffeine metabolism

## Abstract

**Purpose:**

The present study aimed to clarify associations between coffee intake and kidney function with consideration of the effect modifications from coffee intake-related genetic polymorphisms.

**Methods:**

This cross-sectional study included 7,468 Japanese participants 35–69 years old (3,953 women: 52.9%) from the baseline survey of the Japan Multi-Institutional Collaborative Cohort study. Coffee intake was estimated with a self-administered questionnaire. Three coffee intake-related single nucleotide polymorphisms (in *AHR* [rs4410790], *HECTD4* [rs2074356], and *CYP1A2* [rs762551]) were selected with reference to previous studies. Estimated glomerular filtration rate (eGFR [ml/min/1.73 m^2^]) and CKD (defined as eGFR < 60 ml/min/1.73 m^2^) were determined.

**Results:**

In participants with a slow metabolizing genotype of rs4410790, eGFR with higher coffee intake was 1.64 ml/min/1.73 m^2^ (95% CI 0.29–2.98) lower than with low coffee intake. For a frequent coffee consumer genotype of rs2074356, eGFR in participants with moderate coffee intake was higher than with low coffee intake. For heterozygous-type rs762551, coffee intake was associated with a lower prevalence of CKD (OR: 0.53, 95% CI 0.33–0.83). Moreover, with the frequent coffee consumer genotype of rs2074356, higher coffee intake was associated with a lower prevalence of CKD (OR: 0.27, 95% CI 0.08–0.78).

**Conclusion:**

Associations of coffee intake with kidney function and CKD may differ across coffee intake-related polymorphisms in Japanese adults. These findings suggest that attention should be paid to heterogeneous associations between coffee intake and kidney function according to genetic polymorphisms. Further longitudinal studies are expected to address causal questions of these associations.

**Supplementary Information:**

The online version contains supplementary material available at 10.1007/s00394-025-03819-2.

## Introduction

Chronic kidney disease (CKD) is a serious public health problem globally [1]. According to a 2017 survey, 697.5 million cases of CKD were reported and 1.2 million people died of CKD [2]. Japan also has a high prevalence of CKD, and the number of cases continues to increase year by year [3]. On the other hand, pharmacotherapies that can protect against kidney dysfunction or restore lost kidney function are currently lacking. Given this situation, modifiable lifestyle factors are key to preventing CKD. For example, a cohort study of 200,000 Taiwanese adults showed that physical activity was inversely associated with declines in estimated glomerular filtration rate (eGFR) and the risk of CKD [4]. In a cross-sectional, multi-ethnic study in the United States, a dietary pattern high in whole grains, fruits, vegetables, and low-fat dairy products was associated with a 20% lower risk of CKD, while non-dairy animal-based food intake was associated with an 11% higher urinary albumin-creatinine ratio [5].

Coffee is an immensely popular beverage around the world, with 1.4 billion cups consumed each day [6]. In Japan, an average of 10.6 cups a week are consumed per person according to a 2022 survey [7]. Many epidemiological studies have reported that coffee intake shows inverse associations with cardiovascular disease, cancer [8], type 2 diabetes [9], metabolic syndrome [10], and metabolically unhealthy phenotypes [11]. Since kidney function is known to be closely related to cardiometabolic risk factors such as hypertension [12] and hyperglycemia [13], coffee could potentially show protective roles against CKD. Although some epidemiological studies have examined relationships between coffee consumption and CKD or kidney function, the results have been controversial [8, 14, 15]. This discrepancy in results may be due to inter-individual differences (i.e., genetic polymorphisms in caffeine metabolism). For example, one prospective cohort study in Italy suggested that the association between higher coffee intake and deterioration of kidney function was modified by a single nucleotide polymorphism (SNP) in *CYP1A2* (rs762551) [16]. However, other polymorphisms related to caffeine metabolism have not yet been examined in studies. Further, East Asian populations including the Japanese have genetic backgrounds and coffee consumption habits that differ from those of European populations. The aim of this study was to assess the association between coffee intake and kidney function in a general Japanese population, taking into account the effect modifications of caffeine metabolism-related polymorphisms.

## Material and Methods

### Study population

The Japan Multi-Institutional Collaborative Cohort (J-MICC) study is a prospective epidemiological study being conducted by 14 universities and research centers throughout Japan to examine gene-environment interactions in lifestyle-related diseases. The details of this cohort have been described previously [17–19]. In the present cross-sectional study, Japanese male and female subjects between 35 and 69 years old who had participated in the baseline survey of the J-MICC study were included (version October 16, 2023 dataset). From the 8,558 subjects with data available for serum creatinine, coffee intake, and genotypes, we excluded those individuals with a previous history of ischemic heart disease, stroke, or cancer, or missing information on these (n = 859). We also excluded subjects with missing data on smoking habits or physical activity, or for whom total energy intake was extremely high or low (≥ 4,000 or < 1,000 kcal/day, respectively) (n = 231). Finally, 7,468 participants were included for further analysis (Supplementary Fig. 1). Among the 7,468 participants included in the analysis, body mass index (BMI) was missing for one individual. To impute this missing value, we applied multiple imputation by chained equations (MICE) using the *mice* package (version 3.18.0) in R (version 4.4.1). Predictive mean matching (*method* = *“pmm”*) was employed to generate five imputed datasets (*m* = *5*), and a random seed was set to 123 to ensure reproducibility. This study was approved by the ethics committees of Fujita Health University (approval no. HG24-002), Tokushima University Hospital (approval no. 466–15), and Aichi Cancer Center Research Institute (approval no. H2210001A) and by each participating institution. All participants provided written informed consent. The study was performed in accordance with the ethical standards as laid down in the 1964 Declaration of Helsinki.

### Questionnaire

All participants were asked to complete a self-administered questionnaire regarding medical history, smoking and drinking habits, physical activity, educational background and frequency of the intake of foods and beverages. Trained staff confirmed the data obtained. Information on coffee intake was obtained regarding two different types of coffee intake, as follows: i) filtered or instant coffee intake; and ii) canned, bottled, or packed coffee intake. The seven possible choices for each item were: “rarely”, “ ≤ 2 cups/week”, “3–4 cups/week”, “5–6 cups/week”, “1–2 cups/day”, “3–4 cups/day”, or “ ≥ 5 cups/day. These responses were converted to cups per week (0, 1, 3.5, 5.5, 10.5, 24.5, and 35 cups/week), then the values from (i) and (ii) were added together to obtain total coffee intake. Based on total coffee intake, as used in previous studies, participants were divided into groups of “ < 1.5 cups/day”, “ ≥ 1.5 to < 3 cups/day”, and “ ≥ 3 cups/day” [11]. Smoking habits were separated into three categories of non-, ex-, and current smokers, and the average number of cigarettes per day and age at the initiation of habitual smoking were reported. Pack-years were calculated by multiplying the average number of cigarettes per day by the number of years smoked and dividing by 20 (one pack). Drinking habits were likewise separated into categories of non-, ex-, and current drinkers, and the frequency and amount of six alcoholic drinks (Japanese sake, shochu, shochu-based cocktails, beer, whiskey, and wine) consumed each time were reported. The ethanol intake (grams per day) of current drinkers was estimated based on the amount of ethanol present in each alcoholic drink. Information on physical activity during leisure time was asked in terms of frequency (five choices, from none to ≥ 5 times/week) and average duration (six choices, from ≤ 30 min to ≥ 4 h) for light-intensity exercise (e.g., walking, hiking) at 3.4 metabolic equivalents (METs), moderate-intensity exercise (e.g., jogging, swimming) at 7.0 METs, and heavy-intensity exercise (e.g., marathon running) at 10 METs. Total physical activity (MET·hours per week) was estimated by multiplying the frequency and total average duration of each intensity of exercise, and summing the results. Information on educational background classified into four categories (≤ 9 years, 10–15 years, ≥ 16 years, and unknown). Daily total energy intake was calculated from the information on dietary habits assessed by a validated short food-frequency questionnaire using an original program developed and validated by the Department of Public Health, Nagoya City University School of Medicine [20–22].

### Coffee intake-related variants

Blood samples were collected at the baseline survey of each study site and stored at − 80 °C after centrifugation. DNA was uniformly extracted from the buffy coat by well-trained medical technologists. Participants were genotyped using the HumanOmniExpressExome version 1.2 platform (Illumina, San Diego, CA) at the RIKEN Center of Integrated Medical Sciences (Yokohama, Japan). Pre-imputation quality control (QC) for samples and SNPs was performed based on the predefined criteria [23, 24]. An imputed dataset was developed based on the 1000 Genomes Project reference panel (phase 3, all ethnicities)[25]. SHAPEIT (ver. 2)[26] and minimac3[27] were used for phasing and imputation, respectively. We used three SNPs with an imputation quality of *r*^2^ > 0.9 or minor allele frequency (MAF) > 0.01. Referring to previous studies, we selected three coffee intake-related SNPs: *AHR* (rs4410790, 7:17284577, genotyped, MAF = 0.377), *HECTD4* (rs2074356, 12:112,645,401, genotyped, MAF = 0.232), and *CYP1A2* (rs762551, 15:75041917, imputed, MAF = 0.362). Genomic locations for the three SNPs were based on GRCh37. A previous study showed that variants near *AHR* (rs4410790 C), *CYP1A2* (rs2472297 C), and *OR5M7P* (rs597045 A) were more strongly associated with higher intakes of caffeinated than decaffeinated coffee intake [28]. Given that other GWAS for coffee intake also identified SNPs in *AHR* and *CYP1A2*, we excluded *OR5M7P* from these three variants. In addition, rs2472297 was replaced with rs762551, another important SNP in the *CYP1A2*, because rs2472297 is almost monomorphic in East Asian population. rs2074356 in *HECTD4* was ethnic-specific suggesting an association with coffee intake in the Japanese population [29]. Although the mechanism is still unclear, our assumption was that this SNP involved in associations of coffee intake and health outcomes.

### Estimating kidney function

Serum creatinine (in milligrams per deciliter) was measured using the enzymatic method at baseline. At some institutes where serum creatinine was measured using the Jaffe method, measured values were transformed to the equivalent value of the enzymatic method. The formula proposed by the Japanese Society of Nephrology was used to calculate eGFR, as follows: eGFR = 194 × serum creatinine^−1.094^ × age^−0.287^ (× 0.739 for women). This formula has been validated in a general Japanese population [30]. CKD was defined as an eGFR < 60 ml/min/1.73 m^2^.

### Statistical analyses

To assess the associations of coffee intake (< 1.5 cups/day, ≥ 1.5 to < 3 cups/day, or ≥ 3 cups/day) with eGFR and CKD prevalence, we performed linear regression and logistic regression analyses. Regression coefficients and corresponding 95% confidence intervals (CIs) were estimated with adjustments for the potential confounders. In Model 1, we started to adjust for fundamental variables such as age, sex (male or female), and research site (Okazaki, Shizuoka, Takashima, Kyoto, Saga, Kagoshima, Tokushima, or Shizuoka-Sakuragaoka). In Model 2, smoking habits (never-, 0–20 pack-years, ≥ 20 pack-years, or unknown), drinking habits (never-, ever-, 0–20 g/day, ≥ 20 g/day), physical activity (quartiles), education level (≤ 9 years, 10–15 years, ≥ 16 years, or unknown), and total energy intake (quartiles) were added to the variables in Model 1. In Model 3, we further incorporated clinical treatments for diabetes, hypertension, and dyslipidemia (yes or no). In Model 4, green tea intake was added to the variables in Model 3. In Model 5, we added BMI (quartiles) to the variables in Model 3. For heterogeneous coffee-kidney associations across genotypes of each variant, we estimated effect sizes after stratifying genotypes using the same models. Sensitivity analyses were performed to confirm our findings in different settings and subgroups such as analysis with grouped genotypes, and analysis in non-drinkers. The threshold for statistical significance was a two-tailed value of *P* < 0.05. All statistical analyses were performed using SAS version 9.4 (SAS Institute, Cary, NC, USA).

## Results

The characteristics of study participants according to coffee intake are shown in Table 1. Participants with higher coffee intake were younger, more likely to be male, smoke more cigarettes, be ex- or current-drinkers, less physically active, and with a higher level of education. Participants with higher coffee intake had significantly less history of diabetes, hypertension, and dyslipidemia. Participants with higher coffee intake were likely to have the C allele at rs4410790 in *AHR* and A allele at rs2074356 in *HECTD4*, respectively. Further, coffee intake differed significantly across research sites.

**Table 1:** Characteristics of subjects according to coffee intake

	Coffee intake (cups/day)			*P*-value
	< 1.5 (n = 2785)	≥ 1.5 and < 3 (n = 2843)	≥ 3 (n = 1840)	
Age (years)^a^	57 (47, 63)	53 (46, 61)	51 (44, 58)	< 0.001
Sex^b^				< 0.001
Female	1455 (52.2)	1649 (58.0)	849 (46.1)	
Male	1330 (47.8)	1194 (42.0)	991 (53.9)	
eGFR (mL/min/1.73 m^2^)^a^	78 (69, 88)	79 (70, 88)	79 (70, 88)	0.016
CKD (eGFR < 60 mL/min/1.73 m^2^)^b^	235 (8.4)	184 (6.5)	95 (5.2)	< 0.001
*AHR* (rs4410790)^b^				< 0.001
TT	1163 (41.8)	1115 (39.2)	657 (35.7)	
TC	1281 (46.0)	1285 (45.2)	879 (47.8)	
CC	341 (12.2)	443 (15.6)	304 (16.5)	
*CYP1A2* (rs762551)^b^				0.503
CC	362 (13.0)	396 (13.9)	265 (14.4)	
AC	1248 (44.8)	1287 (45.3)	839 (45.6)	
AA	1175 (42.2)	1160 (40.8)	736 (40.0)	
*HECTD4* (rs2074356)^b^				< 0.001
GG	1666 (59.8)	1687 (59.3)	925 (50.3)	
GA	957 (34.4)	984 (34.6)	785 (42.7)	
AA	162 (5.8)	172 (6.1)	130 (7.1)	
Total energy intake (kcal/day)^c^	1669.8 (1489.8, 1900.8)	1646.8 (1475.4, 1852.6)	1676.4 (1499.4, 1920.1)	< 0.001
Leisure-time physical activity (MET-h/week)^a^	6.8 (1.3, 17.9)	6.5 (0.9, 17.9)	5.1 (0.4, 15.8)	0.032
Green tea intake^b^				
Almost none	358 (12.9)	437 (15.4)	375 (20.4)	< 0.001
≥ 1 cup per week	582 (20.9)	555 (19.5)	351 (19.1)	
≥ 1 cup per day	1845 (66.3)	1851 (65.1)	1114 (60.5)	
Education level (years)^b^				< 0.001
≤ 9	319 (11.5)	209 (7.4)	126 (6.9)	
10 ~ 15	1788 (64.2)	1882 (66.2)	1175 (63.9)	
≥ 16	662 (23.8)	736 (25.9)	531 (28.9)	
Unknown	16 (0.6)	16 (0.6)	8 (0.4)	
Smoking habit (pack-years)^b^				< 0.001
0	1788 (64.2)	1744 (61.3)	821 (44.6)	
> 0 and < 20	406 (14.6)	445 (15.7)	343 (18.6)	
≥ 20	536 (19.3)	604 (21.3)	633 (34.4)	
Unknown	55 (2.0)	50 (1.8)	43 (2.3)	
Drinking habit^b^				< 0.001
Non-drinker	1202 (43.2)	1093 (38.5)	693 (37.7)	
Ex-drinker	54 (1.9)	42 (1.5)	40 (2.2)	
> 0 and < 20 g/day	852 (30.6)	1110 (39.0)	659 (35.8)	
≥ 20 g/day	677 (24.3)	598 (21.0)	448 (24.4)	
History of diabetes^b^				< 0.001
Never	2607 (93.6)	2730 (96.0)	1745 (94.8)	
Current or Past	178 (6.4)	113 (4.0)	95 (5.2)	
History of hypertension^b^				
Never	2196 (78.9)	2368 (83.3)	1602 (87.1)	< 0.001
Current or Past	589 (21.2)	475 (16.7)	238 (12.9)	
History of dyslipidemia^b^				0.002
Never	2281 (81.9)	2393 (84.2)	1587 (86.3)	
Current or Past	504 (18.1)	450 (15.8)	253 (13.8)	
Research site^b^				< 0.001
Okazaki	352 (12.6)	393 (13.8)	211 (11.5)	
Shizuoka	648 (23.3)	722 (25.4)	420 (22.8)	
Takashima	158 (5.7)	181 (6.4)	80 (4.4)	
Kyoto	243 (8.7)	375 (13.2)	212 (11.5)	
Saga	743 (26.7)	531 (18.7)	408 (22.2)	
Kagoshima	277 (10.0)	244 (8.6)	180 (9.8)	
Tokushima	136 (4.9)	238 (8.4)	229 (12.5)	
Shizuoka–Sakuragaoka	228 (8.2)	159 (5.6)	100 (5.4)	

The Kruskal–Wallis test was used for continuous variables

The chi-squared test was used for categorical variables

*eGFR* estimated glomerular filtration rate, *CKD* chronic kidney disease, *MET* metabolic equivalents

^a^Median (25%, 75%)

^b^Number (%)

**Fig. 1:**
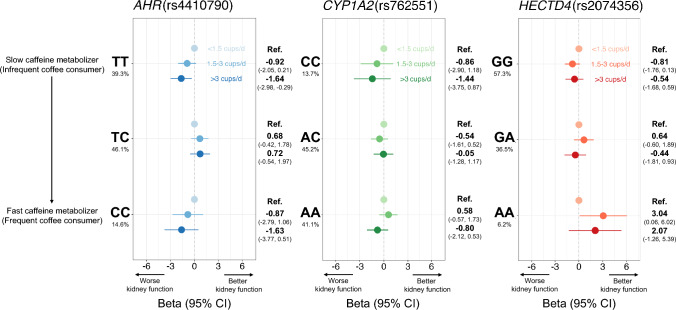
Association between coffee intake and estimated glomerular filtration rate (eGFR) according to genotype in rs4410790 of *AHR*, rs762551 of *CYP1A2*, and rs2074356 of *HECTD4*. Points and error bars depict β values and 95% CIs, respectively. Linear regression to estimate associations between coffee intake and eGFR were performed after adjusting for age, sex, research site, total energy intake, physical activity, educational attainment, smoking habits, drinking habits, clinical history of diabetes, hypertension and dyslipidemia. eGFR: estimated glomerular filtration rate; β: regression coefficient; CI: confidence interval

Figure 1 and Supplementary Table 1 show the associations between coffee intake and eGFR (continuous trait: lower value reflects decreased kidney function) according to genotype in rs4410790 of *AHR*, rs762551 of *CYP1A2*, and rs2074356 of *HECTD4*. In all participants, coffee intake was not associated with eGFR (≥ 3 cups/day, Model 3: β − 0.57, 95% CI − 1.41 to 0.26). In rs4410790 of *AHR*, we found heterogeneous associations of coffee intake with eGFR across genotypes. In participants with TT (slow caffeine metabolizers), eGFR with higher coffee intake was 1.64 ml/min/1.73 m^2^ (Model 3: 95% CI 0.29 to 2.98 ml/min/1.73 m^2^), lower than that with low coffee intake, but no such associations were evident in participants with CT or CC genotypes. For the AA genotype in rs2074356 of *HECTD4*, eGFR in participants with moderate coffee intake was 3.04 ml/min/1.73 m^2^ (Model 3: 95% CI 0.06 to 6.02 ml/min/1.73 m^2^) higher compared with low coffee intake. In grouped genotypes, the association between coffee intake and eGFR was not observed (Supplementary Table 2).

**Fig. 2:**
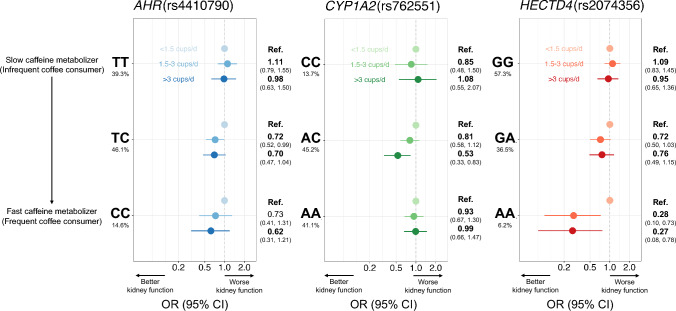
Association between coffee intake and chronic kidney disease (CKD) according to genotype in rs4410790 of *AHR*, rs762551 of *CYP1A2*, and rs2074356 of *HECTD4*. Points and error bars depict odds ratios and 95% CIs, respectively. Logistic regression to estimate associations between coffee intake and eGFR were performed after adjusting for age, sex, research site, total energy intake, physical activity, educational attainment, smoking habits, drinking habits, clinical history of diabetes, hypertension and dyslipidemia. CKD: chronic kidney disease; OR: odds ratio; CI: confidence interval

Figure 2 and Supplementary Table 3 show the associations between coffee intake and CKD (binary trait) according to genotype in rs4410790 of *AHR*, rs762551 of *CYP1A2* and rs2074356 of *HECTD4*. Higher coffee intake was marginally associated with a lower prevalence of CKD among the total participant cohort with an odds ratio (OR) of 0.79 (≥ 3 cups/day, Model 3: 95% CI 0.60 to 1.03). To check heterogeneous associations of coffee intake and CKD prevalence across genotypes, we performed logistic regression analysis after stratification. For rs4410790 of *AHR*, the OR of CKD prevalence in participants with moderate coffee intake was 0.72 (Model 3: 95% CI 0.52 to 0.99) for the TC genotype. In those with high coffee intake, the OR was 0.70 (Model 3: 95% CI 0.47 to 1.04) for the TC genotype and 0.62 (Model 3: 95% CI 0.31 to 1.21) for the CC genotype. Moreover, for the TC + CC genotype, in participants with moderate coffee intake, the OR was 0.74 (Model 3: 95% CI 0.56 to 0.98), and in those with high coffee intake, the OR was 0.69 (0.49 to 0.97) (Supplementary Table 4). For rs762551 of *CYP1A2*, CKD prevalence was lower among those with higher coffee intake only in the AC genotype, with an OR of 0.53 (Model 3: 95% CI 0.33 to 0.83), with no such differences in CKD prevalence according to coffee intake in each of the other genotypes of rs762551. In participants with high coffee intake, the OR was 0.67 (Model 3: 95% CI 0.46 to 0.96) for CC + AC genotype (slow-metabolizing) and 0.74 (Model 3: 95% CI 0.55 to 0.98) for AC + AA genotype (fast-metabolizing) (Supplementary Table 4). For rs2074356 of *HECTD4*, with respect to our analysis of eGFR, higher coffee intake was associated with lower CKD prevalence among the AA genotype with an OR of 0.27 (Model 3: 95% CI 0.08 to 0.78).

Main results were consistent in the analysis restricted to nondrinkers (Supplementary Tables 5 and 6). Moreover, additional analysis was performed adjusting for green tea intake, which correlated inversely with coffee intake (Supplementary Fig. 2). Analysis adjusting for BMI was also performed, since obesity is strongly related to the risk of CKD [31]. The results showed little alteration with adjustment for green tea intake (Model 4 in Supplementary Tables 1–6) or BMI (Model 5 in Supplementary Tables 1–6). Analysis according to types of coffee was performed in total participants. A lower prevalence of CKD in intermediate canned/bottled/packed coffee intake and CKD was observed (Supplementary Table 7).

## Discussion

This study evaluated relationships between coffee intake and kidney function in more than 7,000 Japanese adults. We hypothesized that coffee intake would not have a uniform impact on kidney function, but rather that influences would differ across genetic polymorphisms involved in caffeine metabolism. Indeed, no clear association was found between coffee intake and eGFR in the whole population, but heterogeneous associations were identified across genotypes of caffeine metabolism-related variants. Similarly, for associations between coffee intake and CKD, a beneficial association was observed for specific genotypes in some genetic variants. These findings were consistent across models adjusted for green tea intake and analyses restricted to nondrinkers. The differences in eGFR in this study were relatively small (i.e., 1.64 ml/min/1.73m^2^), but this resulted in a single genetic polymorphism, and larger differences in eGFR may be observed when following a polygenic hypothesis. Our results suggest that caution should be employed when interpreting associations between coffee intake and kidney function for entire populations.

Coffee is one of the most popular beverages throughout the world, with ~ 1.4 billion cups consumed daily [6]. This beverage contains a very large number of chemicals and a growing body of scientific evidence is being accumulated on associations between coffee intake and health outcomes [32, 33]. Most studies have reported beneficial effects of coffee intake on various health outcomes, such as hypertension [34], obesity [35], and dyslipidemia [36]. Regarding kidney function, while recent Mendelian randomization studies have suggested that coffee intake could provide benefits [37–39], associations between coffee intake and kidney function from observational studies remain contentious [8, 40–45].

. This attempt could add new avenues to interpreting evidence on the coffee-kidney axis. In recent prominent reports from US populations [16], coffee intake was suggested to have deleterious effects on albuminuria among slow caffeine metabolizers (AC + CC genotypes of rs762551), but no significant association between coffee intake and albuminuria was found among fast metabolizers with the AA genotype. Given that caffeine-metabolizing capacity could be determined by genetic polymorphisms, such heterogeneous associations need to be investigated across different races/ethnicities. Accordingly, the finding that genotype reveals a heterogeneous renoprotective effect of coffee consumption in East Asians is also valuable.

*AHR*, a gene located on chromosome 7, encodes a receptor involved in the regulation of several genes related to xenobiotic metabolism, including *CYP1A1* and *CYP1A2*, which play roles in processing caffeine. A genetic variant in *AHR* (rs4410790) was first reported in the genome-wide association study (GWAS) of coffee intake with a variant between *CYP1A1*-*CYP1A2* (rs2470893) [46]. Specifically, rs4410790 has shown a notable association with coffee intake, where individuals carrying the T allele tend to consume less caffeine, suggesting a potential impact on caffeine sensitivity or tolerance. For this genetic variant, we observed the results to be in concordance with our original hypothesis. In summary, kidney function among individuals with the fast-metabolizing genotype (CC) is not affected by or show beneficial effects from coffee intake, while the slow-metabolizing genotype (TT) shows a slight strain on kidney function from caffeine intake. The results suggested that higher coffee intake in the TT genotype (slow metabolizer of caffeine) may result in caffeine remaining in blood and then contributing to declines in kidney function.

*CYP1A2* is a gene encoding the enzyme cytochrome P450 1A2, which is involved in metabolizing caffeine. Genetic variations in *CYP1A2* can influence the rate of caffeine metabolism, categorizing individuals into “fast” or “slow” metabolizers. This metabolic variability seems likely to affect individual preferences for coffee and other caffeine-containing products [47]. Previous GWASs have reported that some variants in this gene are associated with coffee intake across diverse ethnic populations [48–50]. In the present study, by stratifying genotypes of *CYP1A2* (rs762551), we examined the associations of coffee intake with kidney function. Counter to our hypothesis that coffee consumption would be beneficial in fast metabolizers (AA) or deleterious in slow metabolizers (CC), we found a lower prevalence of CKD in the heterozygous type (AC). There are some plausible explanations for this result. The first is a difference in genetic architectures for coffee intake between East Asians and Europeans. Referring to a previous GWAS including East Asians and Europeans, *CYP1A2* was one of the top signals in Europeans, whereas *ALDH2* and *AHR* were the top two genes in East Asians. Indeed, in Japan, β-values for coffee intake in a GWAS were 0.213 in rs4410790 (*AHR*) and 0.354 in (*ALDH2*), but − 0.084 in rs58806801 (*CYP1A2*) [50]. Given these findings, *CYP1A2* seems less important for caffeine metabolism in East Asians. In fact, another meta-analysis including only Europeans supported an association between rs762551 and coffee intake [51]. Another explanation is that rs762551 has a different effect on metabolic traits in East Asians compared with Europeans. This may be related to the first reason. As described above, among slow metabolizers (AC + CC), higher coffee consumption was associated with worse kidney function in an Italian population [16]. On the other hand, in a Taiwanese population, higher coffee consumption was associated with a lower risk of hypertension only among AC and CC genotypes [52]. To compare these prior results of rs762551, we stratified AA and AC + CC genotypes, showing a lower OR for CKD prevalence with higher coffee intake among AC + CC genotypes (OR 0.67, 95% CI 0.46–0.96). This finding is in line with findings from the Taiwan Biobank (East Asians) [52]. Those papers only provided comparisons between the AA genotype vs. the AC + CC genotypes, and unfortunately could not determine which inheritance patterns are behind the associations of coffee intake with metabolic traits. In the present study, we tried to find associations between coffee intake and kidney disease after stratifying all genotypes (AA, AC, and CC in rs762551). As a result, the protective association was only observed in heterogenous (AC) genotypes of rs762551. A plausible mechanism may be the preferable balance between caffeine clearance and accumulation of beneficial caffeine metabolites such as paraxanthine, theophylline and theobromine [53]

*HECTD4* is a gene encoding the E3 ubiquitin protein ligase, which is crucial in protein degradation and cellular signaling, particularly by catalyzing the final step in the ubiquitination process. Two GWASs including East Asians found the SNP rs2074356 within *HECTD4*, located in the 12q24.12–13 region, was strongly associated with habitual caffeine consumption [29, 54]. The A allele of this variant is common in East Asians but rare in Europeans, Africans, and Americans. This SNP is in strong linkage disequilibrium with SNPs on chromosome 12q24, which have shown associations with drinking behavior in Asian populations [55, 56]. However, surprisingly, associations between rs2074356 and coffee intake were found to be independent of smoking and alcohol consumption effects [29]. Although the biological pathways remain unclear, *HECTD4* may independently impact caffeine-related behaviors and broader metabolic processes. In this study, among individuals with the AA genotype, higher coffee intake may have beneficial effects on kidney function that are absent in other genotypes. We also confirmed that associations in AA genotypes were still consistent after excluding post- and current-drinkers (Supplementary Tables 5 and 6). This result suggests that the association of coffee intake with kidney function or CKD in AA genotypes of rs2074356 was not confounded by alcohol consumption. *HECTD4* could be a specific gene for caffeine metabolism in East Asians and might modulates the effects of coffee intake on health outcomes.

Some plausible mechanisms have been suggested for protective roles of coffee in kidney function. For example, bioactive compounds in coffee such as caffeine and its metabolites, and trigonelline may attenuate nephrotoxic effects of oxalate-induced epithelial-to-mesenchymal transition of renal tubular epithelial cells [57, 58]. Chlorogenic acid may attenuate kidney fibrosis through the regulation of TLR4/Nuclear Factor (NF)-κB-mediated oxidative stress and inflammation [59]. Further information is awaited regarding the relationships between the potential roles of these bioactive compounds in coffee and the heterogeneous associations between coffee intake and kidney function due to genetic polymorphisms seen in the present study.

As for the types of coffee, there was no such difference in the results between filtered/instant coffee and canned/bottled/packed coffee, although a lower prevalence of CKD was observed in intermediate intake of canned/bottled/packed coffee (Supplementary Table 7). In general, canned/packed/bottled coffee has less bioactive compounds including caffeine [60]. Since rapid coffee intake stimulates the sympathetic nervous system and may increase blood pressure [61], moderate intake of bioactive compounds included in coffee may be better suited to the protective impact of coffee on CKD.

Because this was a relatively large and geographically distant multicenter study, the findings can be generalized to Japanese populations. However, several limitations to this study must be discussed. First, the study design was a cross-sectional, which is possible for reverse causality due to no temporal difference between an exposure and an outcome. Thus, cautions are needed to interpret our results. In the current situation, conducting a Mendelian randomization study is difficult for coffee intake and kidney function stratifying by genotypes for genes related to caffeine metabolism. Second, this study did not consider the metabolism of chemicals in coffee other than caffeine, such as chlorogenic acid. Future studies are expected to take into account more complex metabolic mechanisms [62]. Third, regarding coffee intake, recall bias is inevitable due to nature of a self-administered questionnaire. Furthermore, in the present study, no clear distinction was made between canned coffee and instant coffee. Therefore, caffeine contents may be varied across products. Fourth, due to a lack of information on the amount of sugar or synthetic sweeteners added to the coffee, we could not consider detrimental effects of these substances on kidney function. Fifth, we selected three SNPs for estimating heterogenous effect of coffee intake. However, future examinations should be considered other genes related to caffeine metabolisms (e.g., *ADORA2A*) and integrated with other genetic variants contained in coffee.

We found heterogeneous associations of coffee intake on kidney function and CKD across genotypes of caffeine metabolism-related genotypes. The present results provide important evidence for personalized nutrition to promote lifestyle modification with the goal of slowing declines in kidney function among East Asians. For example, for slow caffeine metabolizers, a personalized message would be to refrain from excessive coffee consumption. From a larger perspective, our study shed light on the importance of heterogeneity rather than population-averaged treatment effects when analyzing the impacts of coffee consumption on health outcomes. Further studies are expected with longitudinal data (e.g., CKD incidence and eGFR during the follow-up period) and stratified Mendelian randomization.

## Supplementary Information

Below is the link to the electronic supplementary material.

Supplementary file1 (PPTX 195 KB)

Supplementary file2 (XLSX 61 KB)

## Data Availability

The data needed to replicate the results of the study are available upon reasonable request to the corresponding author and after approval by all the participating institutions.

## Abbreviations

CKD: Chronic kidney disease
eGFR: Estimated glomerular filtration rate
SNP: Single nucleotide polymorphism
J-MICC: Japan Multi-Institutional Collaborative Cohort
BMI: Body mass index
MICE: Multiple imputation by chained equations
QC: Quality Control
MAF: Minor allele frequency
OR: Odds ratio
CI: Confidence interval
*AHR*: Aryl hydrocarbon receptor
*CYP1A2*: Cytochrome P450 1A2
*HECTD4*: HECT domain E3 ubiquitin protein ligase 4
GWAS: Genome-wide association study
NF: Nuclear factor
