# Supplementary material for: Coffee intake, genetic variants, and chronic kidney disease: a cross-sectional analysis of the Japan Multi-Institutional Collaborative Cohort (J-MICC) study
Source: Eur J Nutr. 2025 Oct 15;64(7):301. doi: 10.1007/s00394-025-03819-2 (PMC12528254; doi:10.1007/s00394-025-03819-2)
Supplement: Supplementary file 1 — Supplementary file1 (PPTX 195 KB) [file 394_2025_3819_MOESM1_ESM.pptx]

## Slide 1
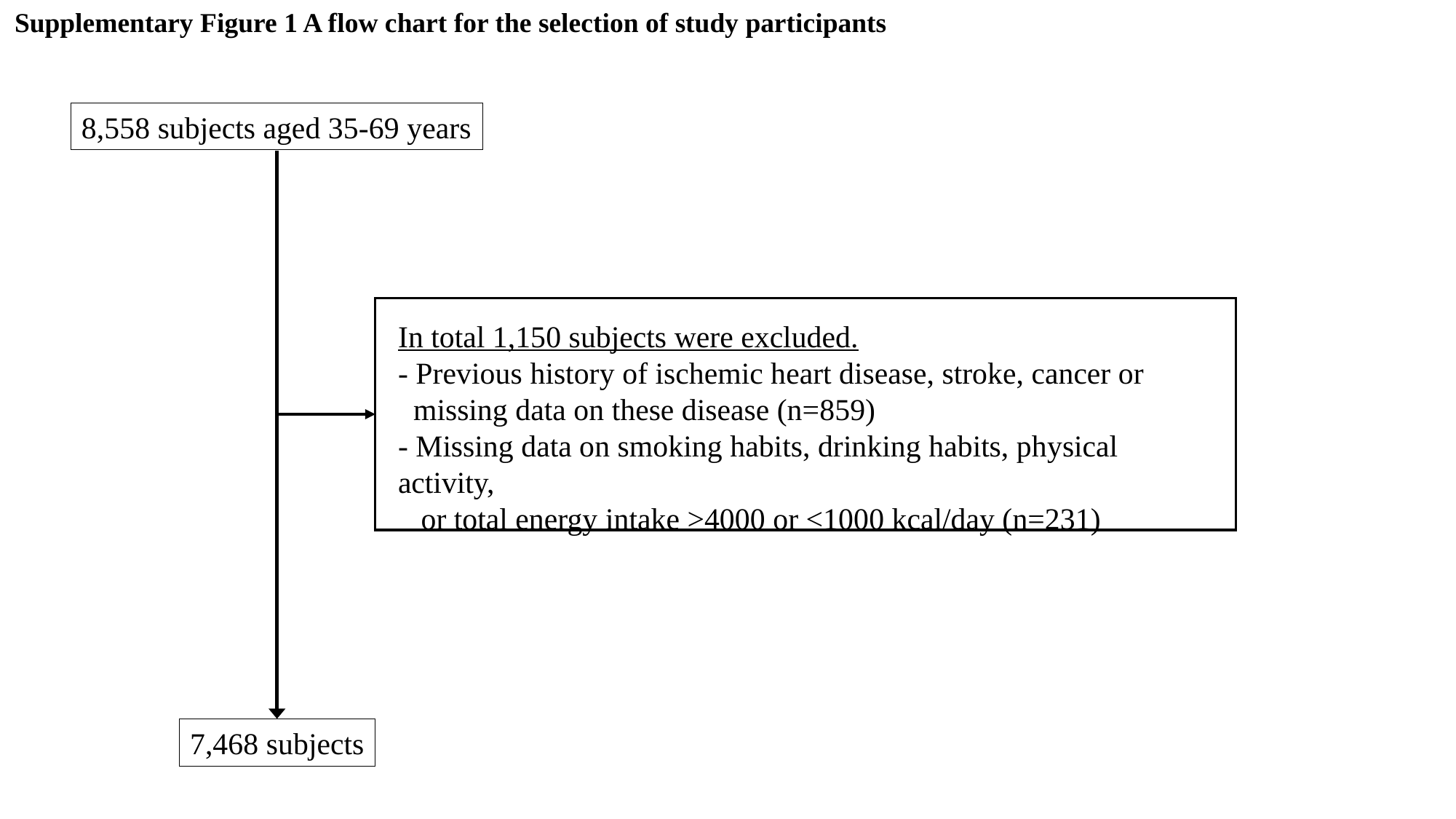

Supplementary Figure 1 A flow chart for the selection of study participants
8,558 subjects aged 35-69 years
In total 1,150 subjects were excluded.
- Previous history of ischemic heart disease, stroke, cancer or
 missing data on these disease (n=859)
- Missing data on smoking habits, drinking habits, physical activity,
 or total energy intake >4000 or <1000 kcal/day (n=231)
7,468 subjects

## Slide 2
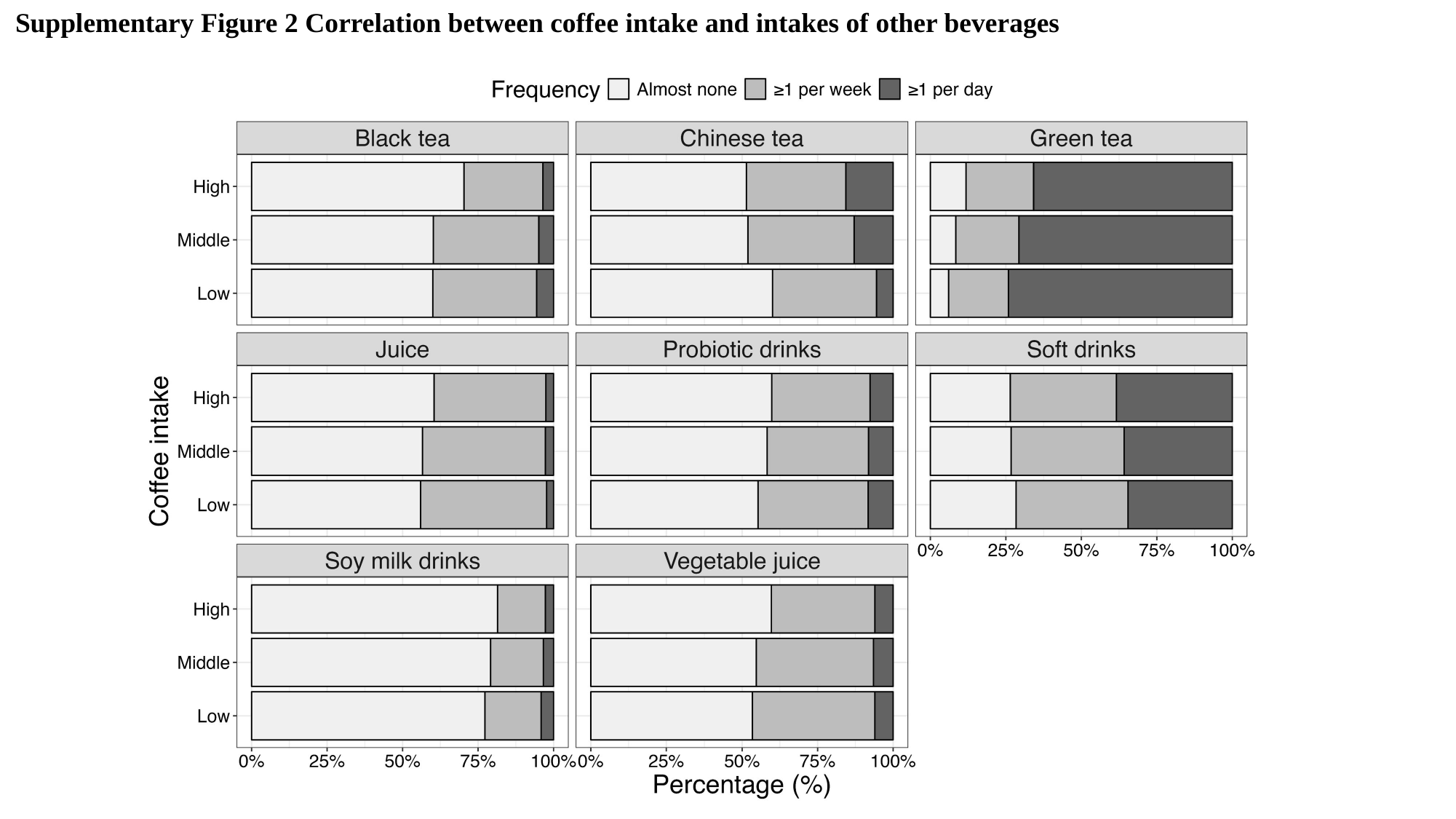

Supplementary Figure 2 Correlation between coffee intake and intakes of other beverages
